# Analysis on the linguistic features of conflict discourse in mathematical cooperation problem solving in China

**DOI:** 10.3389/fpsyg.2022.945909

**Published:** 2022-09-20

**Authors:** Jingbo Zhao, Tingting Song, Xiaoying Song, Yuanmeng Bai

**Affiliations:** Key Laboratory of Data Science and Smart Education, Ministry of Education, Hainan Normal University, Haikou, China

**Keywords:** mathematical problem solving, collaborative learning, conflict talk, discourse analysis, linguistic features

## Abstract

Classroom teaching is a kind of social activity system. Thus, as a form of classroom learning, collaborative problem solving has a strong social attribute. It is extremely common to choose the conflict discourse in the context of cooperation. The verbal characteristics of the conflicting discourse level in cooperative mathematics problem solving directly affects the cooperative learning between students and the classroom teaching of teachers. This article focuses on the overall linguistic characteristics of conflict discourse in solving cooperative problems and the discourse style and language characteristics of the three stages of conflict discourse. The main research conclusions are as follows: (1) The classification of language features of conflict discourse includes extreme summaries, negation, discourse markers, and so on. Among them, the frequency of Indexical 2nd-person pronouns is the highest. (2) The language expressions at the “initial stage of conflict” include Explanatory statement Negative response, instruct refuse and Seditious inquiry Confrontational answer. The language shows the characteristics of using emphatic words or phrases, negative words, imperative sentences and so on. Meanwhile, rebuttal questions, direct responses, explanations, and negative avoidance are the main forms language expressions at the “conflict stage.” It also exhibits the verbal characteristics of rhetorical questions, negative comments, and direct negation. Lastly, topic-shifting, compromise, third-party intervention, and one-sided wins are the linguistic expressions at the “end of conflict.” The language features are the appearance of tone relaxation and language easing, and the conflict ending utterances reflect cooperation.

## Introduction

Cooperation is an important driving force behind the development of human society, and cooperation skills have become one of the most essential qualities for citizens the 21st century. As an important teaching method, various countries have paid increasing attention to cooperative problem-solving in recent years. Students start by realizing social interaction in the process of problem solving, which subsequently improves cooperative cognition. As early as the beginning of the 21st century, the United Nations Educational, Scientific, and Cultural Organization (UNESO) regarded teamwork and problem-solving skills as essential qualities for citizens of the 21st century (OECD, 2020). In 2013, the PISA 2015 Cooperative Problem Solving Framework Draft published by the International Student Assessment Project introduced the Collaborative Problem Solving (CPS) test for the first time (Lin, 2016). “China’s Compulsory Education Mathematics Curriculum Standard (2011 Edition page11)” pointed out: “Experience the process of cooperating and communicating with others to solve problems.” The latest 2022 edition (page 1) also pointed out: “Help students develop the habit of independent thinking and the willingness to cooperate and communicate.”

This article mainly studies the verbal characteristics of the conflicting discourse in cooperative mathematics problem solving. It refers to Scott’s (2002) language classification of conflicting discourse in combination with the language characteristics of middle school students in the process of group cooperation in a Chinese classroom environment to identify the characteristics of conflict words and their distribution trends in the process of cooperative mathematics problem solving to lay a foundation for the further analysis of the impact of speech and distribution trends with different characteristics on cooperative problem-solving. Conflict discourse is a catalyst to help solve cooperative problems (Cazden, 2004). Effective conflict in the classroom can prompt group members to re-examine their initial opinions, promote the verification of a student’s point of view, effectively strengthen students’ motivation to obtain information, and enhance students’ motivation to learn. At the same time, it can encourage learning to actively communicate with others and improve interpersonal communication skills. Conflicts caused by different cognitions among students are very beneficial for them to acquire real knowledge. Solving the conflict in the process of discussion drives the further development of students.

At the same time, Conflict discourse is the normal state of group members’ discourse choice and negotiation in the context of cooperation. Studying Conflict Discourse in students’ cooperative problem solving will help group members further grasp the rules of verbal communication and better understand the power of the team in cooperative learning; Teachers can understand the dynamic process of students’ discourse conflict according to the occurrence, development and ending process of conflict discourse, grasp the interaction law, and provide targeted intervention guidance for group cooperative learning, which has important theoretical and practical significance.

## Literature review

### Status quo of research on cooperative learning based on problem solving

In 1926, some scholars discussed the cooperative problem solving (Alschuler et al., 1977). Cooperative problem-solving integrates the two capabilities of problem solving and cooperation. Since the 1980s, there has been more and more research on problem-solving, especially in the field of education in the United States and Australia. There is a deep interest in this field (Goos and Galbraith, 1996). Since the 21st century, team-based problem solving has been increasingly recognized as having a vital impact on society. Enterprises and society have paid more and more attention to the power of teamwork as well as how interpersonal relationships and the problem-solving abilities of staff are related. In 2013, the PISA 2015 Collaborative Problem Solving Framework Draft included the cooperative problem solving test as part of their test content, with problem solving ability being an important marker of mathematical ability. The problem solving ability has been valued by the United States (Li and Cao, 2019), Finland (Yu and Cao, 2017), and other countries that have achieved good results in the PISA test. In the newly announced PISA 2021 math test framework, the definition of mathematics literacy is clearly defined, with mathematics communication and problem solving an important part of it (Cao and Zhu, 2019).

Yang and Wang (2019) racked and studied the dynamics of cooperative problem-solving research on the international front and came to a series of important conclusions. At present, cooperative problem-solving mainly focuses on the following six hot topics: distributed cognition, cooperative learning, co-building scaffolding, experimental teaching, peer guidance and chemistry. The relatively new research content in the field of mathematics education currently includes multidisciplinary research, early childhood education, and green chemistry research. It is more common about holistic cognition and sequential cognition in the research of cognitive style, and there are some new development trends in this research field. In terms of the cognitive approach, the theory of situational cognition has gradually shifted to the theory of distributed cognition; in terms of education, it has shifted from a single subject to multiple cooperation; in terms of content, it has shifted from traditional subjects to emerging subjects (Yang and Wang, 2019). In China, the research on cooperative problem solving mainly focuses on the macro level, such as the evaluation of cooperative problem solving ability (Hao and He, 2019), the cultivation of cooperative ability, and the model of cooperative learning (Cao and Bai, 2018).

### Research on conflict discourse in mathematical cooperation problem solving

In the process of cooperative problem solving, conflict is inevitable. This conflict usually manifests in the form of “cognitive conflict,” which is caused by different views on the problem rather than other forms of social conflict such as emotional conflict (Shi and Du, 2008). Johnson claims that conflict occurs when “the two parties in the communication hold different views or opinions, and they want to reach an agreement (Zhang, 2020).” The purpose of cooperative learning is to solve problems and for team members to reach a consensus. Team members use various forms of interaction to achieve a new balance among themselves in the form of conflict and negotiation.

The cognitive construction process of cooperative learning is divided into two stages: the individual knowledge construction stage and the cooperative joint structure stage (Liu and Wang, 2008). The stage of individual knowledge construction is the process by which individuals compare their own mental model with the presented material and with other mental models after being presented the learning materials. The cooperative joint structure stage can coordinate differences and is a process of refinement, discussion, and conflict (Wang, 2004). Researchers Berkowitz and Gibbs (1983) divided discussion into expressive and processing discussions before conducting a coding study on it. Alexopoulou and Driver (1997) proved the dual nature of conflict in cooperative learning and concluded that “arguments are composed of other effective types of expressions.”

To sum up, the current international research on conflict discourse mainly includes theoretical research on the definition and function of conflict discourse. The empirical research mainly focuses on the structure and language characteristics of conflict discourse, but most of them are conducted from the perspective of daily conversation. However, there is still a certain research space to explore the types and styles of conflict in classroom teaching from the perspective of mathematics classroom based on real corpus. Research on the conflict and negotiation process of cooperative mathematics problem solving is our focus. This article mainly analyzes the conflict language characteristics in cooperative mathematical problem solving, and empirically studies the conflict and negotiation events in the context of mathematical problem solving based on the existing research.

### Theoretical framework

Social interdependence theory is an important theoretical basis of cooperative learning. It mainly discusses the action efficiency, internal psychological process, interaction mode and results of individuals when they interact in cooperative and competitive social situations. In 1935, Kurt Kafka, the founder of Gestalt psychology in Germany, first put forward the view of “the integrity of group dynamics.” In 1949, the disciple of Lewin, Dodge, developed his theory and proposed two types of positive and negative social interdependence, which directly affected the interaction mode and psychological process of both sides of communication. Then the Johnson Brothers, Dodge’s disciples, constructed a systematic “social interdependence theory” based on their social interdependence theory, forming a series of operable procedures, As shown below:


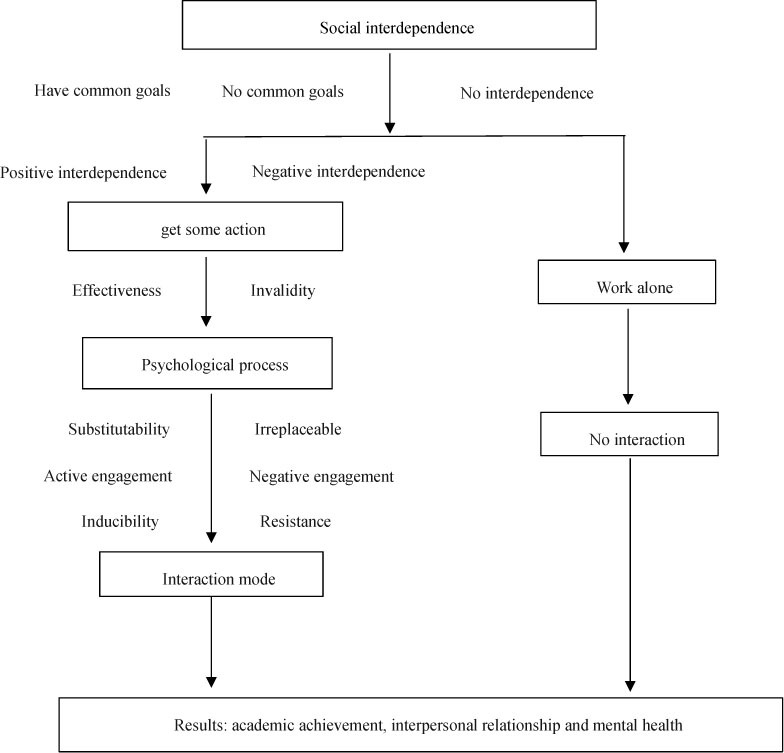


In the process of cooperative learning, positive social interaction should have a good performance in both action and emotion. Conflict is common in cooperative situations. No matter positive or negative interdependence, different individuals will conflict about how to achieve the goal of reciprocity. Facing such a problem, we should face the conflict directly, trust and help each other, and deal with the conflict constructively through the circular system thinking mode of “difference coordination unity.” In the process of positive interaction, individual self-education and self-improvement, so as to complete self transcendence and finally realize self-worth.

In short, from the content of cooperative mathematics problem-solving research, cooperative learning, cognitive interaction, social interaction, peer guidance, etc. are currently the hot issues in the international research on Cooperative problem-solving. At present, the relatively new research contents in the field of mathematics education include multidisciplinary integration research, education at an early age of children, and cognitive style research. There are more holistic cognition and sequential cognition, and there are some new development trends as a whole. In terms of cognitive approach, it gradually turns from situational cognitive theory to distributed cognitive theory; In the aspect of educational subject, from single subject to multiple cooperation; In terms of problem content, it has shifted from traditional disciplines to emerging disciplines. The research on conflict and negotiation in the process of cooperative mathematics problem solving has a certain research space. Based on the existing research, this paper empirically studies the conflict and negotiation events in the context of mathematical problem solving. Based on the analysis of the existing literature, it is found that there are few studies on the types of Conflict Discourse in classroom teaching, especially in the process of group cooperative problem-solving learning. In mathematics education research, the Conflict Discourse Research of cooperative learning is still a blank. Through this research, we can deeply analyze the social interaction in the process of students’ cooperative problem-solving, and then further analyze its impact on the effect of cooperation.

## Research design

### Research questions

Existing research mainly analyzes students’ “cooperative problem-solving ability” from the perspective of assessment ability, but there have been few instances of interactive research on cooperative problem solving in classroom learning, especially in the mathematics classroom. Therefore, this topic will focus on the following issues: (1) What are the overall linguistic characteristics and distribution trends of conflicting discourse in solving mathematical cooperation problems? (2) What are the language expression methods and language characteristics at the three stages of “conflict initiation, conflict and negotiation, and conflict end” in solving mathematical cooperation problems?

### Participants

The research data of this paper comes from the Sino Australian cooperation project “The Social Essentials of Learning (SEL): An experimental investigation of collaborative problem solving and knowledge construction in mathematics classrooms in Australia and China,” Specifically, it comes from the 2018 national general topic of the 13th 5 years plan of China’s Educational Science (BHA180157): “empirical research on cognitive interaction and social interaction and their relationship in middle school students’ cooperative problem solving,” Researchers have been involved in the collection and collation of project data since 2018. All the participants are Chinese students, these students get used to group work in their everyday mathematics classroom learning. In the collected video data, the video data of four people’s cooperative problem-solving is selected as the data source, because the participation of the four-person cooperation group is relatively high, and the conflict discourse phenomenon is relatively prominent.

The data of this study used a total of 67 groups in eight classes of grade one in L middle school and Y middle school in B city. The group with blurred image and sound and the group with more than 4 people were removed. 48 groups were used in this study.

**FIGURE 1:**
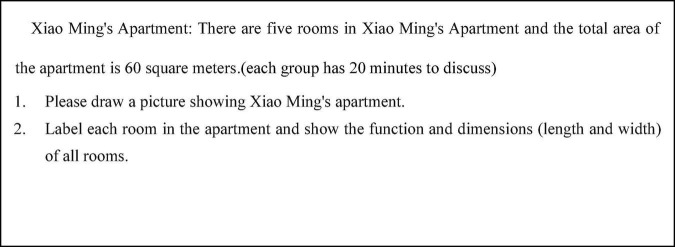
The math problem of “Xiao Ming’s Apartment.”

A study by Bruxelles and Kerbrat-Orecchioni (2004) pointed out that the distinguishing feature of multi-person conversation is “alliances” where people with the same opinion will unite to refute another person’s point of view, which is more likely to cause conflict. The themed task of the four-person group is: “Xiao Ming’s Apartment” (Figure 1).

In this study, the 48 groups with no or little intervention by the teacher was selected as the research object. In this state, the verbal interaction between group members is free and real.

### Research methods

This research focuses on text analysis, and the research steps are as follows: First, the collected video data is text-transcribed to obtain the most basic text data and build the corpus of this research. Secondly, the corpus is further analyzed after using Python software to segment the text. The specific operations are as follows:

Adopting the combination of the retrieval function of the corpus retrieval software Antconc and the retrieval and screening of Excel to realize the analysis of text data, so as to realize the collection and sorting of the characteristics of the hedge sudden language, and then use the jieba in Python to segment the corpus, and use the antconc software to count the word frequency. The python program is as follows:


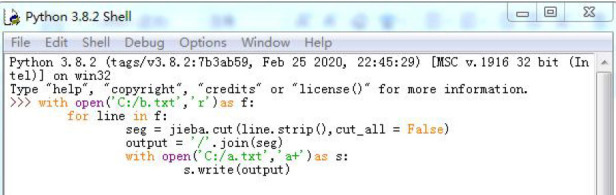


Specific analysis methods also include classroom video observation and case analysis.

Classroom video observation method takes real-time video as a technical means, and has unique advantages in analyzing the speech behaviors of teachers and students in classroom teaching. It can repeatedly observe and record the target characteristics, and analyze the speech behaviors of students scientifically and systematically through group and individual comparative comprehensive research. For different research problems, pre class interviews with teachers and students were conducted before and after the data were collected in the class, mainly to examine the understanding and participation of teachers and students in cooperative problem-solving activities. After all the preparatory work is completed, based on the recording classroom and video equipment, collect and summarize the data of students’ communication audio, video, task list and so on in the process of solving cooperative problems, and carry out coding analysis.

Case analysis method, also known as case study method, is to study specific individuals, units, phenomena and themes, collect relevant data, determine research cases, sort out and analyze the generation and development process of research objects, analyze internal and external factors and mutual relations, and strengthen a more in-depth and comprehensive understanding of the problem. When analyzing the Conflict Discourse Structure in the argumentation process, this study mainly adopts case studies, selects representative focus cases for analysis, and makes a comprehensive and systematic in-depth study on the conflict in the argumentation process in cooperative learning.

### Construction of coding system

When dividing conflict events, we should start from two aspects: task conflict and relationship conflict, and mainly consider three aspects. First, during the discussion, the panel members had different views; Second, the dialogue among the group members is confrontational; Third, the group members have strong emotional expressions when the conflict occurs. Conflict events mainly have three links: initial stage of conflict, Conflict and Negotiation stage and the end of conflict stage. When dividing conflict events, we should pay attention to whether these three links are complete. In particular, the end of conflict in the third link has a variety of forms, including stalemate, compromise, concession, successful negotiation, transfer conflict, etc (Table 1).

**TABLE 1:** Conflict stage and coding.

Conflict stage	Student	Speech characteristics	Code
Initial stage of conflict	S2 S3 S1	Multiply by what, equal to 60? tagging. First think, first think wide. First think about the possibilities of length and width	M1 M2 M3
Conflict and Negotiation stage	S2 S1 S3 S1 S2 S1 S4 S1	2,3 It’s impossible to multiply by 60. It’s impossible to be 2 m wide. If it’s at least 5 m or 10 m wide, right? If it’s 10 m wide, it’s 12 m long. There are five rooms. Five rooms, yes, first of all, if he, if the length is calculated according to the integer solution, then 10, if the width is 6, the length is 10, right, if the width is 8, can 8 be divided? It can be done. 8 can be divided. Don’t you think it’s too square. 15.5	M4 M5 M6 M7 M8 M9 M10 M11
The end of conflict stage	S2 S1 S2 S1 S2 S4 S2 S1	In fact, the formula is better. Let’s use the 10. 10 and 6 are better, the smaller the difference, the better Yes, the smaller the difference, the better. The bigger the difference, the bigger you think it is. Yes But it can’t be too different. It’s absolutely impossible to be thin. Then 6 and 10. 6 and 10, 8 and 7.5 are the best. Width is 6. Yes, you two draw 6 and 10, and we draw 7 and 15.	M12 M13 M14 M15 M16 M17 M18 M19

## Results

### The overall linguistic features of conflict discourse in mathematical cooperation problem solving

Verbal conflict is the main form of conflict in the process of cooperative problem solving. This paper uses the collected video of 48 teams (each team consists of four people) engaged in cooperation problem solving as a corpus database to analyze the verbal characteristics of conflicting language in cooperative problem solving. The classification of discourse features in this article is based on the work of Scott (2002). The language classification of Scott’s conflict discourse is divided into 12 different types. On this basis, this article combines the language characteristics of middle school students in the process of group cooperation and constructs the language characteristics and distribution trends of conflict words in the process of middle school students’ cooperative mathematics problem solving in the classroom environment of our country. We then further analyze the influence of different characteristics of speech and their distribution trends on the effectiveness of cooperative problem solving.

#### Classification of conflict language features

Based on the prominence of the conflict, Scott (2002) classified the verbal characteristics of conflicting discourse into twelve categories: extreme generalizations, negative forms, discourse markers, emphasis, turn-taking, discourse fluency markers, second-person pronouns, modality vocabulary, repetition, question sentence, turn length, and topic avoidance (As shown in Table 2).

**TABLE 2:** Classification of language features of conflict discourse.

Classification	Expressions of conflicting discourse
Extreme summaries (Absolutes)	All, anyone, anything, anywhere, all, every, every person, everything, no matter where, never, no one, no, no place, impossible, must, absolute, sure, certain, definite, only
Negation	No, wrong, false, can not, no way, not at all
Discourse markers	But, now, ok, then
Emphatics	There are indeed, many, more, most, real, true, for example, in case, if
Floor bids	Let me come to him/her/us + verbs (for example, speak), wait a moment
Flow	
Indexical 2nd-person pronouns	You, all of you, yourself, your selves
Modals	Possibility: be able to, can, maybe, probably Necessity: must, should Predictive: shall Semi-modal words: have to do something
Repetition	Restatement of words and sentences
Questions	Interrogative sentence
Turn length	In number of words per turn
Uptake avoidance	Avoidance of previous topic

This study is based on Scott’s classification of language features of conflict words. Through the preliminary analysis of the research object, combined with the speech features of Chinese middle school students in cooperative mathematics problem solving, a speech feature classification suitable for conflict words in this study is constructed. It is mainly embodied in 10 types: extreme generalizations, negative forms, discourse markers, emphatic words, turn-taking words, second-person pronouns, modal words, repetition, rebuttal questions, and topic avoidance. We further combine the conflict fragments in the corpus to determine the analysis unit, determine the similarity pattern in the use of language features in the problem task environment, and analyze its meaning and main feature words.

##### Absolutes

“Everyone, anyone, anything, anywhere, every, every, everybody, everything, no matter where, never, no one, no, no place” etc. are common extreme generalizations in students’ cooperative learning. When the words “every,” “all,” and so on appear in the students’ words, it means that the students are very confident in their words and believe that all situations are under their control, and hope that all group members are able to accept their own opinions. In the follow-up discussion, they will continue to emphasize these points. Such extreme generalizations are often refuted by others because each student’s experience and knowledge level are inconsistent and they will have different views on different situations, which may lead to conflicting discourse.

**Fragment 1:**

S3 Brother, how did you draw it?
S1 It is drawn crooked.
S2 Actually, you can do…
S3 **All** your overall straight lines are drawn crookedly.
S1 Because I watched it backwards.
S2 No, they can actually have a three-dimensional one.
S1 **Does not.**
S2 You can take a look first.
S1 Paper is too small.

Extreme summaries are a form that often appears in cooperative problem solving. As in the above two segments, S3 proposed that “all” straight lines were drawn crookedly, and S1 retorted “No.” Because the third party S2 intervened to let S1 draw one first, it ended the conflict between S1 and S3. As far as the conflicting parties are concerned, extreme generalizations can often stimulate one of the communicators to think about whether there are other possibilities in the plan, and to a certain extent can promote the divergence of the communicator’s thinking.

##### Negation

Negative forms are a main way of causing conflicting discourse, which appears in the form of “no, inability, wrong, not necessary” and other expressions. The negative form is the most direct rebuttal. It shows that the two sides of the communicator are on two sides that are obviously opposite, and they hold two completely different views on the same topic. When the negative form appears, the tone is often stronger. Based on the face theory, the refuted party may feel that his face is “threatened” and will further refute and emphasize his own point of view, which escalates the conflict. If the rebutted party does not conduct another rebuttal but instead asks for the other party’s opinions and negotiates with the other party, then the conflict may be eased as in the following dialogue:

**Fragment 1:**

S4 His house is small, you can see that it is big as a whole, so why do you think our house is so small. Do you know why the house is small? It’s because of the furniture.
S1 Yes, yes, there are still messes in the house.
S4 This is because it is empty.
S1 Then this living room, the cloakroom must be in front of the living room?
S4 **It is wrong, the cloakroom shouldn’t be here.**
S1 Where can the cloakroom be? Is the cloakroom in the bedroom? You wipe it, I painted it first.
S4 **It is not used.** For example, this is the living room, this opens a door, this is the door, this is the door, and this is the cloakroom.

The negative form expresses disagreement with the other party’s point of view. S1 proposes that the cloakroom is in front of the living room but S4 directly denies it. S1 does not refute it further but instead asks for S4’s opinion and proposes to draw first. S4 denies it again and then puts forward its own ideas. In this process, S4 is in a dominant position who hopes that the other party will accept his/her own point of view and draw according to his/her own ideas, which is why he/she used negative words at the beginning of the sentence. In addition to expressing a clear negation, the tone used for opposition is also stronger, expressing a clearer attitude of the individual.

##### Discourse markers

Sun and Fang (2011) conducted a comprehensive study on the types and functions of Chinese discourse markers and divided Chinese discourse markers into at least 17 types according to the word segmentation in this research, in the cooperative problem solving, the students most commonly used discourse markers such as “right, then, but” and so on. When discussing a topic, the two parties will often respond with “right,” “yes,” and so on, which can play a role in connecting the dialogue and making the dialogue more coherent. After the communicator is approved or affirmed, they will further discuss their views in a more harmonious atmosphere; “Then” can play the role of supplementary explanation to develop the topic further and promote a new argumentation process; “but” can play the role of “contrast” or “turning.” Both parties can think and discuss from multiple levels and aspects, making the discussion process more comprehensive and efficient.

**Fragment 1:**

S2 In fact, the square is better, let’s use the 10, 10 and 6 are better, the smaller the difference, the better.
S1 **Yes,** the smaller the difference, the greater the difference you think the bigger it is.
S2 **Yes.**
S1 However, the difference should not be particularly large.
S2 Let’s choose 10 m in length, 6 m in width.
S4 6 and 10, 8 and 7.5 are the best.
S2 It is 6 m wide.
S1 **Yes,** you draw 6 and 10, then you two draw 6 and 10, and we draw 7 and 15.
S4 I feel special square.
S2 **Yes,** let’s see who is better, and then design it yourself.

After S2 put forward his own ideas, he got S1’s affirmative answer “Yes.” The two parties in the communication reached a preliminary agreement, and then further discussed and improved from the same point of view, proposed their ideas, and negotiated with each other in a harmonious discussion atmosphere. Discourse markers can keep the dialogue connected in meaning and promote efficient discussions between the two parties, which have a certain promoting effect on the final formation of a high-quality plan.

##### Emphatics

It is used to emphasize one’s own point of view, mainly in the form of “does, many, more, most, true, true, for example, such as, if, true” and other verbal expressions. In the process of group discussion, students often emphasize their own opinions to convince others to get on the same page. Whether other people respond to their own opinions determines whether the advocates further emphasize their opinions. If they get a positive response, then the two sides of the communicator reach a preliminary agreement. If a negative response is received, the advocate will re-examine his point of view and give examples to support his claim.

**Fragment 1:**

S4 10 meters in the kitchen is a bit too long, so don’t talk about it first, and finish the question first.
S2 can only be 2 and 5. **Really,** my sister’s house is very long and very narrow.
S4 Does 3 × 3 work?
S2 3 × 3, 9.
S4 let’s still choose 3 m in length and 3 m in width.

The use of emphasized language may escalate or ease the conflicting discourse, which depends on the attitude of the two parties in the communication. S2 claimed that “length and width can only be 2 and 5” before giving an example of “my sister’s house is…” to supplement his own point of view. S4 did not directly refute S2’s claim but he tried to negotiate instead. The attitude and tone of both parties were not strong, and the discussion atmosphere was more harmonious, which promoted the initial consensus of the two parties.

##### Floor bids

The words used to fight for the right to speak are mainly expressions such as “let me/he/she/we come + verbs (for example: speak, say)” “wait a moment” and so on. The fight for the right to speak often occurs when the communicator does not get a response after putting forward an idea, or when the topic is changed and he wants to demonstrate his point of view again. It may also happen when you were unsatisfied with your first argument, did not express it clearly, and want to attract the attention of others again in the follow-up. It is more in line with the thinking development level of students in this age group who want to defend their sovereignty and status, are self-centered, and do not consider whether others are making speeches. The language skills of students of this age group are not perfect, and there may be unclear expression which renders them unable to persuade others. After thinking again, students will want to further add to their arguments. However, a sudden interruption of other people’s speech may cause confusion in the scene and cause conflict.

**Fragment 1:**

S4 draw one less.
S1 **wait a moment.**
S4 Paint a little less, and paint this for the toilet.
S1 Let me take a look. What is 22 minus 8?

The topic of S4 is “Where is the toilet painted and how big is the painting,” but S1 interrupted S4’s topic “Wait a moment” “What is 22 minus 8?” They are not on the same topic - S1 is still immersed in the previous topic and wants to attract the attention of others. At this time, the discussion is in a chaotic situation, which will cause further conflict between the two parties. If one of the parties does not give up on their topic, then the discussion will not proceed, which will have a certain impact on the quality of the final plan.

##### Indexical 2nd-person pronouns

The Indexical 2nd-person pronouns mainly include “you” and “yourself.” In the process of group cooperation, when the other person’s point of view is inconsistent with one’s own point of view, the Indexical 2nd-person pronouns are often used to highlight the inconsistent point of view, to show that one has an opposing point of view, and to emphasize one’s own point of view. The tone of the Indexical 2nd-person pronouns is sometimes strong, and there are cases where one party in the communication orders the other party about. Students at this age have strong self-awareness and self-esteem, and the commanding tone may cause students to think that they are in a disadvantaged position. As such, they are more likely to raise conflicts in the discussion process to defend their status.

**Fragment 1:**

S3 Oh. Why don’t **you** separate it?
S1 Do **you** think that painting is appropriate?
S3 then **you** close the window.
S1 study room must be the quietest.
S4 **You** will soon make the wall thicker.

**Fragment 2:**

S4 What is it now, do you know? An apartment belonging to the villa category.
S1 If it gives us three layers, right? Three-storey apartment. Each floor is 60 square meters.
S4 three sixty eight.
S1 OK, who fills it in, who fills it in.
S2 Let her draw later.
S1 **You** paint, **you** paint. It’s okay to use mine.
S3 No, I haven’t finished it yet.
S1 OK, **you** don’t do it. Okay, I’ll do it.

In the first segment, S3 asks “Why don’t you?”, S1 responds with “Do you think. is appropriate?” to express their own rebuttal. To which S3 proposes “You close the window” with a strong tone. Both parties of the communicator use Indexical 2nd-person pronouns which are inconsistent with each other. If one of the parties insists on their views, the conflict will escalate further. In Fragment 2, “Let’s draw” and “None of you do it” highlights the problem of small group labor. The use of Indexical 2nd-person pronouns expresses dissatisfaction and helplessness with the division of labor. The discussion atmosphere of the group subtly descends into disharmony, which has a certain impact on the quality of the final plan.

##### Modals

Modal verbs mainly express possibility (can and may), necessity (must and should), prophecy (about to), while semi-modal words express the need to do something. Modal verbs expressing possibility are weaker than modal verbs expressing necessity, indicating that the speaker is not very sure, which leaves room for one’s own expression and others’ response. Modal verbs and semi-modal verbs that express necessity and prophecy are stronger, indicating that the speaker has a very positive attitude and wants others to accept his own point of view. Words that express possibility show a willingness to negotiate, which makes the discussion atmosphere more harmonious and can promote initial consensus among the communicators. Modal verbs that express more necessity may cause strong rebuttal from the other party and escalate the conflict. It is not conducive to the formation of the final plan.

**Fragment 1:**

S2 The toilet **must** be written smaller, the toilet, really, someone really has a two-square-meter toilet. Then write a larger bedroom, write a smaller kitchen, and write a larger living room. Make a plan. How many now? How about five square meters?
S4 It **should** be in circles.
S2 **may** have two toilets. toilet.
S4 **should** have a living room.

This is the discussion of the team members when planning the function and size of the room. S2 proposed that “the toilet must be smaller” and expressed his affirmative attitude. S4 replied that “it should be in a circle,” which supplements S2’s point of view and affirms their own view. S2 once again proposed that there are two toilets possibly with a weaker tone than before, indicating that he is now more open toward the opinions of others. To which S4 again replied “should have a living room” to indicate that he is more affirmative and his desire for a response from others. The use of modal verbs can promote the further deepening of the discussion and allow the team members to form a more satisfactory plan in the continuous argumentation.

##### Repetition

Repetition mainly refers to the repetition of words and sentences, with the purpose of emphasizing one’s own point of view and attracting the attention of others. If there is no response, the communicator will re-emphasize it until the other person responds to his point of view. If a negative response is received, the communicator will look for a new basis again, re-emphasize it, and the discussion will be further advanced.

**Fragment 1:**

S1 You don’t need to draw special, just mark each room, why use it, five rooms, three rooms, two rooms, **two rooms and one living room,** **one kitchen and one bathroom.**
S3 **Two rooms and one hall, one kitchen and one bathroom.**
S1 It is just right, **two rooms, one hall, one kitchen and one bathroom,** just right.
S3 **Two rooms, one living room, one kitchen and one bathroom,** almost the same.

This segment is a discussion on the function of the room. S1 first proposes “two rooms and one hall, one kitchen and one bathroom,” S3 gives a repeated response before S1 and S3 repeat it again. The two emphasized the plan many times, highlighting their affirmative attitude toward the plan, hoping to get the attention and approval of others. In line with the language expression characteristics of students at this stage, they hope to emphasize their views through constant repetition before finding new grounds for argumentation.

##### Rebuttal questions

One of the group members raised rhetorical questions about the views or speech behavior of the other party. Such rhetorical questions often have a provocative tone which can easily lead to conflict. After one party in the communication raises a rebuttal question, the other party will feel that his views have been questioned and actively defend his position, which will cause conflict. At this time, it may turn from task conflict to relationship conflict, accompanied by emotional dissatisfaction of students. If both parties in the communication keep repeating rebuttal questions, the discussion will be put on hold, which delays the process and is not conducive to the formation of the final plan. If the rebuttal question is based on the task itself, it will encourage both parties to consider their views more comprehensively and demonstrate them better, which can promote discussion to a certain extent.

**Fragment 1:**

S2 If it is me, if I paint, just draw a three-dimensional one.
S3 1:100, 1:10,000, 1:10,000.
S1 You can’t draw three-dimensional.
S2 **Why can’t draw three-dimensionally?**
S1 Just can’t draw three-dimensional.
S3 Just can’t draw three-dimensional, because yours is a floor plan, **how do you draw three-dimensional?**
S1 This is a floor plan.
S4 **Isn’t it the floor plan I just drew?**

This clip is a conflict between the team members about the drawing scale of the plan. S1 put forward “Can’t draw three-dimensional” to which S2 asked “Why can’t draw?” The tone was provocative because S2 thought that he could draw three-dimensional pictures. This triggered S1 and S3’s rebuttal “You just can’t draw because it is a floor plan” to which S4 again refuted “What I just drew is a floor plan.” The rebuttal questions that appeared in the dialogue caused emotional dissatisfaction among other group members, which led to conflict. The use of rebuttal questions is more in line with the thinking development level of students of this age. Based on their existing learning and life experience, they already have their own way of thinking and will remain suspicious of the opinions of others. In the process of discussion, rebuttal questions can develop students’ dialectical thinking to a certain extent.

##### Uptake avoidance

When a conflict occurs and the two sides are deadlocked, the team members will try to avoid the conflict entirely. The main manifestations of this include topic change, silence, and third-party intervention. Changing the topic is the most commonly used method. When the two parties in the communication cannot reach an agreement and one of them does not want to cause an argument, that party will choose to initiate the next topic to divert attention. When the other party feels that the dispute has not received a response, it automatically ends the argument. The use of topic avoidance can promote the process of group discussion to a certain extent, avoid the emotional dissatisfaction of group members due to disputes, and have a certain positive effect on the final formation of a high-quality plan.

**Fragment 1:**

S4 6 and 10, 8, and 7.5 are the best.
S2 The width of is 6.
S1 Yes, you draw 6 and 10, then you two draw 6 and 10, and we draw 7 and 15.
S4 **I feel special square.**
S2 Yes, let’s see who is better, and then design our own design.
S1 Draw the frame first. You can use the pencil. I don’t have a scale. Who can lend me? I don’t have a scale. Thank you.

When S4 put forward “I think it’s a special square,” other team members ignored it and continued to determine the “overall area of the room” based on the opinions of other team members. If only one group member puts forward a different opinion, the topic will often be avoided and the discussion continued, which has a positive effect on maintaining the feelings between the group members.

#### The overall distribution trend of language features of conflict discourse

The general trend analysis of the language characteristics of conflict discourse takes the lexical level analysis as the object. So seven types of conflict discourse are selected for analysis. Based on the statistical results of computer software analysis, the linguistic feature statistics of the vocabulary level of conflict discourse in group cooperation are shown in the following table (Table 3).

**TABLE 3:** Statistical table of language features at the lexical level of conflict discourse.

Language features at the lexical level	Frequency
Extreme summaries	483
Negation	886
Discourse markers	529
Emphatics	68
Floor bids	16
Indexical 2nd-person pronouns	1154
Modals	622

The above data shows that at the word level, the Indexical 2nd-person pronouns occurred at the highest frequency. In the process of interaction, “you” was used the most, the use of these words not only shows that the individual has a clear direction as well as the opposition and distance between oneself and the other party, but also accuses the other party (Connor Linton, 1989); followed by negative form words. The application of negative form is mainly to express dissent and negate the other party’s point of view. The application of these words is most likely to cause conflicts between group members; The third is the application of modal words such as can, possibly, must, should, will, etc. The application of these words reflects some of the negotiating significance of the team members in the problem-solving process; There is also the application of discourse markers such as right, then, but, and so on. The analysis found that Floor bids occurred the least. In actual classrooms, it is possible that some students compete for the right to speak but do not necessarily use floor bids.

In summary, although the general trend of language features in conflict discourse is a more general overview of the language features of group cooperation, it can also reflect some of the students’ language preferences in this process, which can serve as reference for teachers looking to intervene in cooperative classroom teaching. In the 1950s, the speech act theory was first proposed by the British philosopher J. L. Austin. After this theory, Zhao (2009) analyzed the conflict discourse and proposed three stages: initial stage of conflict, Conflict and Negotiation stage and the end of conflict stage. The following will analyze the language characteristics from these three aspects.

### Linguistic features of “the initial stage of conflict” in mathematical cooperative problem solving

This section mainly analyzes the initial stage of conflict discourse and uses fragments to explain its language expression and characteristics.

#### Language expression in the initial stage of conflict discourse

##### Explanatory statement ↔Negative response

In verbal interaction, the description of objective facts is a more frequent verbal act. In the course of the presentation, the two interacting parties will respond accordingly to their different views on the content of the presentation.

**Fragment 1:**

S1 **Our door is usually on the corner, right? let’s confirm the door first, this corner.**
S2 **No, you don’t need to draw this one inside.**
S1 No, you have to draw like this.
S2 You have to calculate the length and width.
S1 No, what you are asking for is the total area. Look, look at me, the door is facing this side, and draw this side. In this way, it will have a small corridor, right? The small corridor is here, so here is the locker, right next to the door is the locker.

The conflicting discourse that begins with the explanatory statement is caused by the speaker’s different views on people and things. According to corpus research, the speaker and responder in conflicting discourse are mainly in the mode of “explanatory expression and negative response.” In the form of narrative, affirmation, appreciation, and approval of the speaker, what is waiting is the direct opposition, denial, questioning, or interruption of the other party’s response, which leads to conflict.

##### Instruct↔refuse

In verbal interaction, the speaker issues instructions, suggestions, requests, and orders to the other party to engage in a certain behavior. After the other party responds with confrontation, rejection, questioning etc., the verbal conflict will begin. The expression of this “instruction rejection” mode occurs in the form of imperative and declarative sentences. E.g.:

**Fragment 1:**

S4 There are only five rooms, so save a bit.
S1 No need to save.

In this dialogue, S4 suggested to save a little while allocating area, and S1 directly rejected it. In the verbal interaction, the caller’s instruction hopes to get an affirmative and accepting answer, and the recipient S1 can either obey the instruction of S4 or refuse. Obviously, in the above case, the recipient refuses to accept the other party’s instructions, which leads to the beginning of a frontal conflict between the two sides.

##### Seditious inquiry ↔Confrontational answer

In verbal interaction, one question and one answer often leads to conflict. Questions are trigger words, and answers are responses to inflammatory or provocative questions from the speaker, as per the following example:

S4 Zhang Yang, what kind of ghost do you paint?
S2 Go! Don’t insult my creativity.

In the above-mentioned conflicts, S4 issued an inflammatory question that was discriminatory to a certain extent. The other party, S2, was not to be outdone, leading to a frontal conflict between the two parties. The inflammatory questioning was also an important reason for conflicting discourse.

#### The linguistic features of “the initial stage of conflict” in conflict discourse

The initial stage of conflict is the origin of the entire conflict process and plays a vital role in the occurrence of conflict. By categorizing the expressions of conflicting discourse in this stage, this article finds that the linguistic characteristics of the “initial stage of conflict” have the following points:

##### The use of accents or phrases

Emphasized words or phrases are used to enhance the degree of expression, which can be adjectives, adverbs, interjections, pronouns, or modal particles. In conflict conversations, both women and men tend to use emphasized words or phrases to enhance their feelings, but women are better at using such words because they have a rich emotional network. Men often use emphasized words or phrases to express their differences. In the initial stage of the conflict, the two parties in the conflict often choose modal particles, such as “ba,” “le,” “of,” “ah,” “ma,” “ah,” etc.; there are also pronouns, such as the first person “I.” When they have a conflicting conversation, they use these words to express their strong emotions.

##### The use of negative words

Most conflicts are caused by disagreements between communicators, and they often use the negative word “no” to express their disagreements. In the initial stage of the conflict, both parties to the conflict usually choose the negative words “no,” “can’t,” “impossible,” and so on. According to the face threat theory, the use of negative words by one of the two parties will inevitably threaten the “face” of the other party, and the conflict will further escalate.

##### The use of imperative sentences

The imperative sentence has the function of being a command, instruction, or warning. If there is “please” before the imperative sentence, it will be regarded as a request. Negative imperative sentences are used to prohibit someone from doing something or to discourage someone from doing something. In dominant interactive networks, imperative sentences are highly used. In the initial stage of conflict, some group leaders will use their authority to use imperative sentences to command group members, which further intensifies the conflict. For example, “You paint, don’t make trouble, you don’t make trouble, take it away,” “You give up,” “You don’t, you don’t,” “The living room must be the largest.”

### Language features of “conflict and negotiation stage” in mathematical cooperation problem solving

In the process of group cooperative learning, the conflict stage is the core part of a conflicting verbal event, and it is also the main part of the conflict from the beginning to the final agreement. After the conflict begins, it usually takes only one or two rounds for the members to reach a consensus, but sometimes it takes many interaction rounds between the group members to reach a consensus.

Based on the analysis of text data, this article mainly discusses the language expression methods and language characteristics of the “conflict phase” of cooperative problem resolution. The language expression of the “conflict stage” mainly includes several types such as the rebuttal questions, the direct response, the explanation, and the negative avoidance.

#### The language expression of “conflict and negotiation stage”

##### Rebuttal questions

In the process of group cooperation problem-solving conversation, when the listener expresses opposition to the speaker’s point of view, provocative counter-questioning is the most common way of expressing conflicting discourse.

This form of rebuttal question can easily lead to re-rebuttal by the speaker, leading to further intensification of conflicts.

**Fragment 1:**

S3 now has four.
S2 four, plus one.
S3 storage room.
S2 Storage room, let me think about it.
S4 balcony.
S1 balcony.
S3 **Does the balcony count? (With a provocative tone)**
S2 Balcony is counted.
S4 Balcony does not count as a room.
S3 Balcony does not count as a room.
S3 Balcony does not count as a room.
S4 It is not a room, it just occupies an area.
S3 Yes, it occupies an area, but not a room.

##### Direct response

In the process of discourse conflict, facing the conflict caused by the speaker, other team members responded in a straightforward manner. Based on the face theory, the straightforward denial or questioning of one party’s point of view further aggravated the conflict.

For example, in the case of “explanatory statement and negative response,” S1 and S2 had a fierce discourse conflict on the issue of “door.” The dialogue was conducted in a straightforward manner such as “No, you don’t need to paint this one,” “No, you have to paint like this,” etc. The attitudes of both sides were very straightforward, which led to further intensification of the conflict between the two.

##### Explanation

After a conflict occurs, in the face of questions and inquiries from the speaker, the listener gives the reason for the objection, which help resolves the discourse conflict. The explanation refers to the dialogue formed by the listener facing the inquiry or question raised by the speaker during the utterance interaction of the group members.

**Fragment 1:**

S4 Isn’t it awkward to put the kitchen next to the toilet?
S2 Not awkward, anyway, it’s not your own home, really.
S1 No, no, the kitchen and the toilet should not be next to each other, the kitchen should be next to the bedroom, and the living room should be next to the kitchen.

When S4 put forward a point: “Is it awkward to put the kitchen next to the toilet?” Both S1 and S2 gave their own opinions and explained their reasons. S2 thinks “not awkward” because “it is not your own home anyway,” which means you can design whatever you want. However, S1 thinks this design is unreasonable, because “the kitchen and the toilet can’t be next to each other,” the kitchen should be next to the bedroom, and the living room should be next to the kitchen.

##### Negative avoidance

In the process of discourse conflict, when the listener disagrees with the speaker’s point of view, he will often adopt an avoidance method to express his negative point of view instead of making a positive reply. There are three main forms of avoidance: intentional avoidance, temporary avoidance, and unintentional avoidance. After the listener avoids the topic, if the speaker insists on turning the topic back, it may lead to an aggravation of the conflict. But on the whole, when one party has entered negative avoidance mode, it indicates that one party wants to end the discourse conflict, which transitions the entire conflict event toward the end.

**Fragment 1:**

S2 wait a minute, one, two, three, four, one more time.
S1 restaurant.
S3 The kitchen should be with the dining room.
S1 restaurant and kitchen, kitchen has not been written yet.
S2 It is enough, the kitchen is 10 square meters, is 60 enough?
S1 The rest is the kitchen.
S3 Why do you want 10 square meters if you don’t eat?
S2 20, 30, 35, It’s still 15 square meters short, almost, the long one is almost the same as the bedroom.
S3 Our home is also a long strip.
S4 yours too.
S2 draw a picture.
S4 draw it.

S2 advocated “10 square meters in the kitchen” to which S3 asked “Why do you want 10 square meters if you don’t eat?” – expressing denial. S2 did not respond to this question directly and said “Draw a picture” instead, which aggravated the conflict no further.

#### The linguistic features of “conflict and negotiation stage” in conflict discourse

This stage is the key stage in the entire conflict. During this process, the conflict between the two interacting parties escalates rapidly, and a large number of conflicting words will be produced, showing strong antagonistic characteristics and possibly even some offensive words. The research in this paper finds that the language characteristics of the “conflict phase” are as follows:

##### Rhetorical questions

Rhetorical questioning is a way of expressing negation through rhetorical questioning using truths or facts with distinctive features to achieve the purpose of strengthening tone, emphasizing semantics, and expressing blame. In the “conflict phase,” both parties will usually use rhetorical questions to express their strong doubts about each other. This will lead to the climax of the conflict where the two parties will refuse to give in to each other, which will escalate the conflict. For example, “Is your house made of walls?” “The row is so full, can you still paint?” “Then what do you say this is?” “A two-meter bedroom, are you still sleeping?”

##### Negative comments

In the process of group cooperative learning, group members’ opinions on the problem are evaluated negatively through negative words, which imply accusations and criticisms from the speaker. In the “conflict phase,” both interacting parties will express their disagreement through the use of negative evaluation terms. According to the theory of adaptation, both parties are neglecting to adapt to the contextual factors which will escalate the conflict and eventually result in a stalemate. For example, “Shut up, you shut up.” “Can you stop being so funny?” “Okay, you.” “Can we still play happily?”

##### Direct negation

Direct negative words are often expressed as negative words “no/not + ……” and other structures. That is, to refute by directly negating the proposition stated by the other party, which is manifested when A says proposition P, and B says¬P. In the “conflict phase,” the two interacting parties will express their own confrontation by directly denying the other’s views, which will escalate the contradiction. For example, “No, I think about it. This bedroom is connected to the living room, because my bedroom is connected to the living room.” “No, it needs a balcony.” “No, like me, the bedroom, the kitchen.” “No, the balcony cannot live in.”

### Linguistic features of “end of conflict” in mathematical cooperation problem solving

In cooperative learning, the ultimate goal of discourse conflicts among group members is to form a unity of viewpoints and achieve the purpose of problem solving. No matter how intense the process of discourse conflict is, the conflict on a certain issue will eventually end. This section mainly introduces the expression methods and language characteristics of the ending stage of discourse conflict.

#### Ways of language expression at the end of the conflict

How the conversation ends in discourse analysis is also an important analysis content. In the discourse conflict of group cooperation problem solving, the group members finally reached a unity of opinions after several rounds of disputes and negotiations, which ended the discourse conflict. The way the group members end their conflict and their language characteristics are the focus of this section.

##### Topic-shifting

When the group members argue about a certain point of view, and the two sides are in a stalemate with each other, one of them tries to terminate the topic and find another way to talk about the topic instead. After a period of arguing between the two parties, one party changes the subject and the conflict ends. This not only preserves the face of both parties in the conflict, but also easily achieved a win-win situation for both parties in the conflict.

**Fragment 1:**

S1 Shouldn’t we list it first, why do we have to draw it first. The house doesn’t have to be square.
S2 We didn’t say to draw a square.
S1 Our opinions cannot be unified at all.
S1 What else is there besides the living room, toilet, and bedroom?
S3 kitchen.
S1 Yes.
S2 What to add, as for the specific range, 1, 2, 3, 4, 5, a total of five.
S1 One more.

S1 and S2 had different opinions on the overall shape of the house at first, and a conflict occurred. Then S1 changed the subject and questioned the functions of the five rooms. The resolution of the previous conflict resulted in a new round of discussions.

##### Compromise

**Fragment 1:**

S2 The toilet must be written smaller. The toilet, really, some people have a two-square-meter toilet. The bedroom should be larger, the kitchen should be smaller, and the living room should be larger. Make a plan. There are a few now. How about five square meters?
S2 This is not enough, these five are so big, plus this, so big, stand on the toilet and take a shower.
S3 10 square meters.
S1 10 square meters are too big.
S3 9.
S2 7 square meters.
S1 Made do with it.

S2 advocates that the toilet should be written smaller, S3 said “10 square meters,” S1 said “10 square meters is too big,” S3 said “9,” and finally S2 said “7 square meters” to which S1 agreed to “make do with it.” The three people had different ideas about the size of the toilet, and finally compromised on “7 square meters.”

##### Third-party intervention

When two parties in conflict are arguing about a certain point of view, the intervention of the third party can play a coordinating role to end the conflict. In many cases, this type of ending plays a regulatory role that can promote the rapid unification of opinions.

**Fragment 1:**

S4 Is your length drawn longer? Is your width drawn longer? Is your width drawn longer?
S3 2 cm. 2.
S4 Shrink it.
S3 You cannot change the number of square meters.
S4 Toilet is actually suitable by this. Then you are directly here, and the bedroom is directly here. I should rely on this.
S4 How big is the bedroom?
S1 Let’s not discuss it yet.

S3 and S4 were in conflict over the length and width of the room, and they also had different opinions on the layout of the room. As a third party, S1 intervened in their conflict and asked them to “not discuss it yet” to resolve the conflict.

##### One-sided win

Compared with the conflict ending method in which participants voluntarily compromise, a one-sided win is achieved when one party conveys a message through verbal expression or physical action and compels the other party to accept his own point of view. Although this ending temporarily ends the discourse conflict, it may also create hidden dangers for the subsequent relationship between the two parties, which leads to the emergence of new conflicts.

**Fragment 1:**

S2 The toilet can be smaller.
S4 Toilet is at least bigger than the balcony, right? Is not it?
S1 The toilet is bigger than the balcony. Our house is 40 or 50 square meters.
S1 Our public toilet, can you manage it?
S3 10 square meters public toilet.

S2 advocated that the toilet should be smaller, while S1 advocated that the toilet should be larger than the balcony, and said “Can you manage it?” The attitude was tough, so other students had to accept his idea. This temporarily ended the conflicting dialogue.

#### The linguistic features of “the end of conflict” in conflict discourse

The “end phase” of the conflicting discourse is ended by the two sides of the conversation changing the topic, making a compromise, or reaching an agreement through negotiation. The adaptation theory put forward by Giles and Powesland (1997) claims that language in interpersonal communication has two pragmatic purposes: harmonyorientation and divergence orientation, and harmony orientation is a basic orientation for group members in cooperative learning. Harmony orientation will affect the choice of speech forms and interaction strategies (Spencer-Oatey, 2000). At this stage, the two parties in the communication pass through the previous conflict initiation and escalation stages, and some of the opinions are agreed while some are still deadlocked. However, both parties will also compromise or evade to end the conflict. The tone of the two parties or one of them is not as strong as before, and they are in a state of wanting to end the argument. According to the theory of adaptation, both parties have the awareness of adapting to the context and no longer overemphasize their own ideas, so that conflicts are gradually resolved. The language at this stage has the following characteristics:

##### Moderation of tone and the appearance of mitigator

The moderation of tone and language are to reduce and weaken the intensity of certain language factors in verbal interaction, and to reduce the risks of interpersonal conflicts and face-threatening behaviors in the interaction, so as to ensure the smooth progress of the interaction (Yu, 2015). At the end of the conflict discourse, in order to achieve the goal of harmony, the two parties in the conflict often use mitigation methods or words such as changing the topic and compromising to alleviate the conflict. Moderation language has the function of realizing effective communication and constructing a clear interpersonal identity. It reflects the strategic and practical characteristics of moderation language. Moreover, moderation language generally follows the basic principle of “face threat” proposed by Brown and Levinson (1987). In the process of discussion and exchange in the cooperation group, the other party’s “face” will also be taken into consideration and compromise is adopted to end the conflict. In this study, the emergence of a moderating tone was mainly accompanied by facial expressions and body language. Members often used “laughing” to ease conflicts.

##### Conflict end discourse reflects cooperation

Cooperative learning is to use students as human resources in teaching to make up for the inadequacy of teachers and to cultivate their cooperative spirit, rather than simply shrinking the self and enlarging the collective. In cooperative learning for the purpose of solving mathematical problems, conflicting discourse is the result of the combined effect of many factors such as context and discourse structure. This stage can best reflect the cooperative tendency of “seeking common ground while reserving differences.” To achieve cooperation tasks, even if there are differences in opinions between the two parties in the conflict, one of the parties will choose to compromise, avoid the topic, or stay silent to reach an agreement with the other party. Through the sharing of resources among students during the interaction of group members. Students have their own subjectivity, the interaction between the subject and the subject using dialogue as a means and understanding as the goal, with the goal of reaching a consensus. The different ways of thinking and different understandings of the problem among the team members make the conflict dialogues constructive.

## Summary

This study mainly analyzes the general linguistic features of Conflict Discourse in Chinese students’ cooperative problem solving, including the classification of conflict discourse language characteristics and the conflict discourse vocabulary level before exploring its distribution trend. The linguistic features of conflict discourse include extreme generalizations, negative forms, discourse markers, emphatic words, turn-taking words, second-person pronouns, modal words, repetition, rebuttal questions, and topic avoidance. Among them, the frequency of Indexical 2nd-person pronouns is the highest; the frequency of negation, extreme summaries, discourse markers, and modals lie somewhere in the middle with little differentiation; the frequency of emphatics and floor bids is the least.

The frequency of language features at the lexical level can reflect the language features of conflicting discourse in the process of cooperative mathematical problem solving. However, there are many factors affecting it, such as social factors, participant status, role, scene, and discourse structure factors such as discourse type. These deep-seated reasons will affect participants’ choice of language forms such as negation, modals, discourse markers and questions.

Secondly, based on the three stages of conflict discourse, the discourse style and language characteristic analysis were carried out respectively. Among them, the language expressions of the “initial stage of conflict” include Explanatory statement ↔Negative response, instruct↔refuse and Seditious inquiry ↔Confrontational answer. The language shows the characteristics of using emphatic words or phrases, negative words, imperative sentences and so on. These types of sentences or phrases will further intensify the conflict. In conflict conversation, both boys and girls tend to use emphatic words or phrases to enhance their feelings, but girls are better at using this kind of words. Boys often use emphatic words or phrases to express their differences, and often use the negative word “no” to express dissent. Some leaders of small groups will use their authority to order group members with imperative sentences, which further intensifies the conflict. Rebutting interrogative, straightforward, explanatory, and negative avoidance type are the language expressions of the “conflict stage.” The conflict between the two sides of communication escalates, resulting in the largest number of conflict words. The language shows a strong antagonism, and some offensive words will appear It also exhibits the verbal characteristics of rhetorical question, negative comments, and direct negation. The use of these types of language will bring the conflict to a climax, and the two parties will be deadlocked. Topic-shifting, compromise, third-party intervention, and one side wins are the linguistic expressions of the “end of conflict.” At this stage, both parties involved in the communication begin to have a sense of compliance, their tone of voice is relaxed, and mutual compromise or concession. As a result, conflicts are gradually resolved, tend to end, gradually reach consensus, and tend to be completed through cooperation.

## Data availability statement

The raw data supporting the conclusions of this article will be made available by the authors, without undue reservation.

## Ethics statement

The studies involving human participants were reviewed and approved by Ethics Committee School of Mathematical Sciences, Beijing Normal University. Written informed consent to participate in this study was provided by the participants’ legal guardian/next of kin. Written informed consent was obtained from the individual(s), and minor(s)’ legal guardian/next of kin, for the publication of any potentially identifiable images or data included in this article.

## Author contributions

JZ: research design, data collection, data interpretation, and wrote the main manuscript text. TS, XS, and YB: data collection and data interpretation. All authors reviewed the manuscript.
